# Intention to seek help for depression and associated factors among residents of Aykel town, Northwest Ethiopia: cross-sectional study

**DOI:** 10.1186/s13033-019-0274-y

**Published:** 2019-03-26

**Authors:** Shegaye Shumet, Telake Azale, Getnet Ayano, Dessie Abebaw, Tadele Amare, Wondale Getnet

**Affiliations:** 1 Department of Psychiatry, College of Medicine and Health Sciences, University of Gondar, Gondar, Ethiopia; 2 Department of Health Education and Behavioral Sciences, Institute of Public Health, University of Gondar, Gondar, Ethiopia; 3 Amanuel Mental Specialized Hospital, Addis Ababa, Ethiopia; 4 Department of Epidemiology, Jimma University, Jimma, Ethiopia

**Keywords:** Depression, Intention to seek help, Ethiopia

## Abstract

**Background:**

Depression is the leading cause of disability at a population level and globally 350 million people are suffering from depression. Despite a high prevalence and serious consequences, people with depression are reluctant to seek help and a large proportion remains untreated. The aim of this study was to assess intention to seek help for depression and associated factors among residents of Aykel town, Northwest Ethiopia.

**Methods:**

This cross-sectional population based study included 832 participants. We used a major depressive disorder case vignette and general help-seeking questionnaire (GHSQ) to assess intention. Study participants selected by multistage cluster sampling technique. Face-to-face interview used to collect data. We performed adjusted multiple linear regression analyses to identify factors for intention to seek help.

**Results:**

The mean score on intention to seek help from any health professionals was 3.72 (SD = 1.23) with a range of (1 “very unlikely” to 5 “very likely”). Majority of the respondents had an intention to visit health professionals to get a remedy for their illness depicted in the vignette. The proportion of those who had an intention to seek help from any health professionals if they were having depression was 71.2%. Favorable attitude towards seeking professional help for depression positively associated with an intention to seek help with (unstandardized β = 0.03, 95% CI (0.01, 0.05), p-value < 0.001). Other factors positively associated with an intention to seek help for depression were age (β = 0.02, CI (0.01, 0.03) p < 0.001), and perceived need of treatment for depression (β = 0.19, CI (0.01, 0.38), p < 0.05). Poor social support was negatively associated with an intention to seek help for depression (β = − 0.39 CI − 0.68, − 0.10, p < 0.05).

**Conclusions:**

The results suggest that over two-third of the respondents reported that they were likely or very likely to seek help from health professionals if they were having depression. Increased age, favorable attitude to depression, and perceived need for treatment were factors for intention to seek help. Interventions focusing on awareness creation and attitude change towards depression are necessary.

## Background

Mental health remains a neglected issue in most developing countries, especially in rural areas [1]. Mental disorders were the leading cause of years lived with disability worldwide. Depressive disorder accounted for 40.5% of DALYs caused by mental disorders [2]. Depression affects people in all communities worldwide [3]. One of the most tragic result of depression is suicide [4]. Over 350 million people affected globally with depression. While depression is present in both gender, it is more common in women than men [5]. Depression decreases as people get older [6]. In Ethiopia, depression contributed 7% of the total disease burden [7].

Negative beliefs and attitude, social norm and past treatment behavior predict low perceived depression treatment need [8]. Most people believed vitamins and special diets were more often rated as helpful and dealing with the problem alone would be helpful [1]. Perceived stigma toward depression also has an impact upon the intention of people to seek professional help [9]. Despite mental disorders are prevalent in worldwide, people have understood depressive symptoms as less serious and not requiring treatment and therefore those suffering from depression are unwilling to seek professional help [10, 11]. So caring for these individuals, particularly in the rural areas left to the families and local healers. Regarding to gender, women exhibited more favourable intention to seek help from mental health professionals than men, likely because of their positive attitudes concerning psychological openness [3]. Access to mental health care in low income countries like Ethiopia remains poor. Currently, in Ethiopia ministry of health is practicing community based mental health services including integration of mental health service at primary health care (PHC) level. Social acceptability in relation to ignorance and belief systems is an impediment to mental health care services in Ethiopia. According to most Ethiopians’ perception, mental illness believed to be affliction by psychosocial stressors and supernatural forces (like demon possessions, bewitchments by an evil spirit, ancestor’s sprit, or evil eye) rated more important causes of mental than physical illnesses [12]. Prayer and home/family care suggested more strongly for treating mental than physical illnesses [12, 13]. Attitude towards mental illness affect people to seek help from health professionals [14, 15]. Most people in Ethiopia use traditional methods for treating mental illness and those who look for a modern treatment do so having tried the local means [16, 17]. Affected individuals or their families often seek help from religious and traditional healers rather than health facilities [7]. Understanding intention of people to seek help is important for early intervention of the burden of depression since intention is a predictor of behavior [15, 18]. However, to the best of my knowledge there is a limited information regarding communities’ intention for depressive symptoms in Ethiopia. Therefore, this study focus on help seek intention and identify associated factors for depressive symptoms among residents of Aykel town.

### Objective

This study aimed to assess intention to seek help for depression and associated factors among residents of Aykel town, North West, Ethiopia.

## Methods

### Study design and setting

We conducted a population based cross-sectional study among adults in Aykel town, Chilga district, Northwest Ethiopia between April and May, 2015. The town has a total population of 18,507.

### Sampling

A multi stage cluster sampling technique was used to sample the community members. Simple random sampling technique was used to select one kebele (an administrative unit of Ethiopia, similar to a ward and consists 5000 people) from total of two kebeles since population is large. Each kebele has ketenas (cluster), which is subdivision of kebele. Ketenas were selected randomly and we were selecting 12 ketenas. From the selected 12 ketenas, every eligible individuals/adults/living in these ketena were interviewed by data collectors until the determined sample size full.

### Measurement

General help seeking questionnaire (GHSQ) adapted from previously used research among young people’s help-seeking for mental health problems in Australia [19].

We used general help seeking questionnaire with case vignette for major depressive disorder to measure intention to seek help. GHSQ is measured with likert type scale ranging from (1) “very unlikely” to (5) “very likely”. “If they were feeling like symptoms described in the vignette how likely is it, they would seek help from health professionals?.” High scores for professional help show a person had a good intention.

#### Depression vignette

A 28-year-old man has been feeling unusually sad and down-hearted for most of the day for over 2 weeks. He doesn’t feel like eating and has lost weight. He can’t keep his mind on his work and his financial income has dropped. He has put off deciding and feels that even day-to-day tasks are too much for him. To him, life feels meaningless, and he doesn’t feel he is worth much as a person. If you were feeling like above case how likely it is, you would seek help from the following people? Please show your response by putting a line through the number that best describes your intention to seek help from each help source.

We used Short Explanatory Model Interview (SEMI) to measure perceived severity, cause and need for treatment. We adapted the instrument from previously used research in London [20]. To examine perceived severity, we asked respondents: “How sever do you think is the illness presented in the vignette?” and the response were mild, moderate, severe and very severe. We assessed perceived need for treatment by questioned:” Do you think this illness requires treatment?” and the response were yes/no.

We used Attitudes Toward Seeking Professional Psychological Help (ATSPPH) to assess attitude towards depression. We adapted from research conducted in Turkey among college students [21]. This instrument has 10 items with a likert scale response which ranges from (1) “strongly disagree” to (5) “strongly agree”. Higher scores reflect more attitudes that are positive.

We measured social support using Oslo 3-items, social support scale and with scores ranging between 3 and 14: 3–8 = poor social support; 9–11 = intermediate social support; and 12–14 = strong social support [22].

#### Family history of mental illness

To examine the family history of mental illness, we asked: “Do you know a family member who had experienced a similar situation, or whether they had ever felt this way to that described in the vignette?”

Items on socio-demographic factors (age, sex, ethnicity, religion, marital status, educational status and occupational status) were adopted from different literatures.

### Data collection

We collected data by four trained data collectors (public health professionals) using the Amharic version of the questionnaire for a month. We designed the questionnaire in English and translated to Amharic, the official language of Ethiopia and back to English, forward and backward translation. The training was on introduction to depression and research methods, interviewing skills, sampling and recruitment and ethical aspects of research.

### Data processing and analysis

We checked all the collected data for completeness and consistency and entered to EPI INFO version 3.5.3 and then exported to SPSS for windows version 20 for analysis. We computed descriptive and simple linear regression analyses to see frequency distribution and to test whether there was an association between the independent and dependent (help seeking intention) variables, respectively. In the current study, the internal consistency for intention to seek help for depression was measured by cronbach’s alpha which was α = 0.79.

We selected factors associated with help seeking intention during simple linear regression analysis with a value of p ≤ 0.2 for further analysis in multivariable regression analysis. We considered variables with p-value as statistically significant.

### Ethical consideration and consent

We obtained ethical clearance from the University of Gondar institutional review board and Amanuel Mental specialized Hospital. Formal letter of permission obtained from Aykel town administration. After explaining the aim of the study and the confidentiality issue, we invite them to take part in the project. We obtained verbal consent from participants after asking” are you voluntary to take part in the study?” and if they were voluntary, they would put their signature for that idea was their own. Confidentiality was maintained by omitting their personal identification.

## Results

About 832 participants took part in the study, with a response rate of 98.3%. The mean age of respondents was 28.7 (SD = 9.5) years and 489 (58.8%) of the respondents were male. Concerning educational status, about one in five either had attended primary education or hadn’t attended any formal education. From the total of participants 407 (48.9%) were single (see Table 1).

**Table 1:** Socio demographic characteristics of intentions to seek help for depression among residents of Aykel town, Northwest Ethiopia 2015 (n = 832)

Characteristic		Frequency	%
Sex	Male	489	58.8
	Female	343	41.2
Age	Mean (SD) = 28.7 (9.5)		
Ethnicity	Amhara	486	58.4
	Kimant	326	39.2
	Tigre	20	2.4
Religion	Orthodox	698	83.9
	Muslim	124	14.9
	Protestant	10	1.2
Educational status	Unable to read and write	66	7.8
	Elementary school (1–8th)	98	11.8
	Secondary school (9–12)	365	43.9
	Diploma	154	18.3
	Degree and above	149	17.9
Occupation		243	29.2
	Government employee		
	Merchant	152	18.3
	Private worker	115	13.8
	House wife	102	12.3
	Student	147	17.7
	Jobless	73	8.8
Marital status	Single	407	48.9
	Married	387	46.5
	Divorced	26	3.1
	Widowed/widower	12	1.4

Out of the total respondents, 123 (14.8%) said, they have a family member with mental illness similar to the situation presented in the vignette. About 363 (43.5% reported poor social support), 389 (46.8%) moderate social support and 81 (9.7%) had strong social support of the respondents.

Regard to perception about depression, only 75 (9%) understand depression was a severe illness and about 668 (80.3%) thought psychosocial factors cause it. Slightly less than half 391 (47.0%) of the respondents perceived that depression requires treatment (see Table 2). The mean score of participants’ attitude towards depression was 38.87 (SD = 4.39) (see Table 3).

**Table 2:** Perception about depression among Aykel town residents, North west, Ethiopia 2015 (n = 832)

Variable	Frequency	Percent
Perceived cause of depression		
Psychosocial	668	80.3
Physical	103	12.4
Spiritual	37	4.4
Other^a^	22	2.6
Perceived severity of depression		
Mild	75	9.0
Moderate	116	13.9
Severe	313	37.6
Very severe	328	39.4
Perceived need of treatment for depression		
Yes	391	47.0
No	441	53.0
Appropriate treatment		
Biomedical	295	35.5
Traditional	65	7.8
Other^b^	31	3.7

Other^a^ = I do not know

Other^b^ = I do not know, fulfilling basic needs, refreshing, doing work

**Table 3:** Responses to the item of attitude subscales among Aykel town residents, Northwest, Ethiopia, 2015 (n = 832)

Items	Strongly disagree		Disagree		Undecided		Agree		Strongly agree	
	Freq	%	Freq	%	Freq	%	Freq	%	Freq	%
If you believed that you were having a depression, your first inclination would be to get treatment of depression	38	4.6	160	19.2	124	14.9	349	41.9	161	19.4
The idea of talking about problems with a professional strikes me as a poor way to get rid of emotional conflicts	114	13.7	285	34.3	106	12.7	225	27.0	102	12.3
If you were experiencing a serious depression at this point in your life, you would be confident that you could find relief in professional	20	2.4	57	6.9	138	16.6	390	46.9	227	27.3
There is something admirable in the attitude of a person who is willing to cope with his or her conflicts and fears without resorting to professional help	25	3.0	84	10.1	61	7.3	367	44.1	295	35.5
You would want to get psychological help if you were worried or upset for a long period of time	14	1.7	23	2.8	37	4.4	469	56.4	289	34.7
You might want to have professional help in the future	15	1.8	72	8.7	135	16.2	401	48.2	209	25.1
A person with an emotional problem is not likely to solve it alone; he or she is likely to solve it with professional help	13	1.6	33	4.0	46	5.5	408	49.0	332	39.9
Considering the time and expense involved in professional help, it would have doubtful value for a person like you	235	28.2	365	43.9	70	8.4	103	12.4	59	7.1
A person should work out his or her own problems; getting professional help would be a last resort	63	7.6	152	18.3	99	11.9	351	42.2	167	20.1
Depression, like many things, tend to work out by themselves	84	10.1	189	22.7	177	21.3	251	30.2	131	15.7

### Intention to seek help for depression

**Fig. 1:**
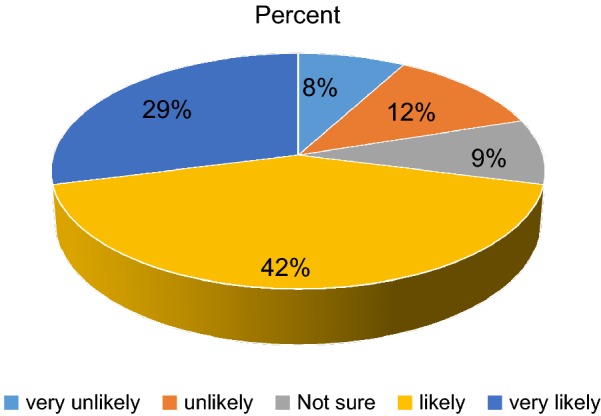
Intentions to seek help for depression and associated factors among residents of Aykel town, Northwest Ethiopia 2015 (n = 832)

Mean score of intention to seek help from any health professionals was 3.72 (SD = 1.23). Implying that the majority of the respondents had an intention to visit health professionals to get a remedy for their illness depicted in the vignette. A close look into each item of the scale also shows that many respondents intended to visit health professionals soon. For instance, the proportion of respondents who said, they were likely or very likely to seek help from any health professional if they were having symptoms described in the vignette was 592 (71.2%). After presenting the depressive vignette we were asking:

“If you were feeling like symptoms described in the vignette how likely is it, you would seek help from health professionals?”. The response were likert type scale ranging from (1) “very unlikely” to (5) “very likely”. High scores show good intention (see Fig. 1).

### Factors associated with an intention to seek help for depression

Simple linear regression analysis of help seeking intention found age, educational status, marital status, social support, perception to the illness (depression) and attitude were significant factors of intention to seek help at a p-value less than or equal to 0.2. We did not enter educational status, gender, ethnicity, religion, perceived severity of depression to the multiple linear regression since p value greater than 0.2. In the multiple linear regression model age, the participant attitude towards seeking professional help, perceiving depression needs treatment, married individuals and moderate and poor social support significantly associated with an intention to seek help for depression (Table 4).

**Table 4:** Simple and multiple linear regression analysis showing significant predictors of help seeking intention from any health professionals for depression among Aykel town residents, Northwest Ethiopia, 2015 (n = 832)

Variables	Crude β (CI)	Adjusted β (CI)
Constant	–	1.65 (0.76, 2.53)
Age	0.02 (0.01, 0.03)	0.03 (0.01, 0.04)^a^
Educational status		
Degree	Ref	Ref
Unable to read and write	0.09 (− 0.27, 0.44)	0.002 (− 0.37, 0.38)
Elementary school (1–8)	− 0.08 (− 0.39, 0.23)	0.15 (− 0.17, 0.47)
Secondary school (9–12)	− 0.15 (− 0.38, 0.08)	0.16 (− 0.17, 0.47)
Diploma	0.06 (− 0.22, 0.34)	0.19 (− 0.08, 0.46)
Marital status		
Single	Ref	Ref
Married	0.01 (− 0.16, 0.18)	− 0.20 (− 0.39, − 0.01)^a^
Divorced	− 0.09 (− 0.58, 0.39)	− 0.25 (− 0.73, 0.24)
Widowed	0.71 (0.004, 1.4)	0.03 (− 0.70, 0.77)
Social support		
Strong social support	Ref	Ref
Moderate social support	− 0.26 (− 0.55, 0.04)	− 0.30 (− 0.59, − 0.10)^a^
Poor social support	− 0.37 (− 0.67, − 0.08)	− 0.40 (− 0.69, − 0.10)^a^
Perceived severity		
Mild	Ref	Ref
Perceiving depression is moderate	0.06 (− 0.11, 0.23)	− 0.24 (− 0.53, 0.05)
Perceiving depression is severe illness	0.02 (− 0.19, 0.24)	− 0.03 (− 0.25, 0.18)
Perceiving need of treatment		
No	Ref	Ref
Yes	0.27 (0.10, 0.43)	0.20 (0.02, 0.38)^a^
Attitude	0.05 (0.03, 0.06)	0.04 (0.02, 0.06)^a^

CI: confidence interval; β: unstandardized co-efficient, ref: reference

^a^Indicates statistically significance factor

## Discussion

This case vignette based assessment of intention to seek help showed that 592 (71.2%) of the respondents had an intention to seek professional help if they were having depression. Of the 832 participants’ mean score of intention to seek help from any health professional was 3.72 (SD = 1.23) with a range of (1) very unlikely to (5) very likely. Implying that the majority of the respondents, 71.2%: 95% CI (68.3%, 74.3%) said they are likely or very likely to seek help after presenting them a vignette depicting an individual experiencing MDD and asking them to report their intention to seek care if confronted with a similar situation. This is similar to the study conducted in China which is 69.6% [23]. It is also similar to a study conducted in Botswana in which participants were likely to seek help from a health professionals for depression [24].

Attitude plays a great role in predicting help seeking intention from a health professional for depression. In this study, attitude was a statistically significant factor for intention to seek help about depression and the finding was supported by a study conducted in Botswana [24] and among Latino immigrants, Mexico [22]. Our result was also similar to New Zealand’s study, with favorable attitudes were related to higher help seeking intentions [25]. The more positive attitude of the participants to depression care, they intent to seek help from professional will be increased. It might be because of individuals with a positive attitude about depression may disclose their illness to health professionals or positive beliefs that professional help is useful which brings a positive attitude.

In this study intention to seek help predicted by age. As age increases health professionals are very important to seek help than family [26]. For young people, the main barriers to seeking help were embarrassment or concern about what others might think [27]. Older age predicted a survey study conducted in England suggest that stronger intention to seek help [28], New Zealand with older adults have a positive attitude to seek professional help for mental illness [25]. As age increase identifying, describing and managing their emotion (emotional competence) may be increased [26]. It might also be because of as age increase depression is not perceived as minor and need the help of health professionals when compared to low in an age in which parents or other adults preferred to seek treatment.

There was a negative association between moderate and poor social support and help-seeking intention. When moderate and poor social support increased, help-seeking intention decreased by 0.30, 0.40 respectively. It might be due to support close to the individual increases attitude and intention to seek help [29]. It might also be due to family members worry could be a factor or influence in whether or not someone would consider seeking help.

There was also a negative association between being married and help-seeking intention and this finding is in contrary to China’s finding [23] in which being married positively associated to seeking help from health professionals. The difference may be in this study married individuals may prefer to seek help from their couple instead of health professionals. The other explanation could be in Chinese study they include the depressed patients.

A unit increase in an intention to seek help from the health professional when participants perceived depression needs treatment. It might be due to people may understand and perceive the illness is very severe and health professionals more helpful than other options. It might be also due to illness causal belief variation. People with physical illness causal beliefs may intent to seek help from health professionals because they may think the problem could not improve by itself spontaneously. As a result, a variety of treatment may need including consulting modern health professionals. This is supported by North-western Ethiopia study on perceptions of mental and physical illnesses [12].

The limitation to this study was that participants did not consider whether or not they have depression. Because participants’ intention to seek help from health professionals might be different when they have depression or do not have depression. The other limitation was the study does not considered perceived stigma and previous help seeking behavior.

The strength of the study was including a relatively large sample size and sampling methods.

## Conclusion

Intention to seek professional help for depression was fairly high considering the socio-cultural characteristics and the belief systems of the community in the study area. Increasing age, holding a favorable attitude towards depression and perceived need for biomedical treatment were the driving forces for intention to seek professional help. On the other hand the perception that one receives poor social support is a barrier to help-seeking persuasive communication to change the attitude of younger adults towards depression may improve help-seeking. For future researchers we recommend that comparative study on intention to seek help among non-depressed and depressed individuals to see their intention to seek help from health professionals. Further increase awareness about depression to community is better to seek help from health institution.

## Abbreviations

CI: confidence interval
DALY: disability adjusted life year
EPI Info: epidemiological information
GBD: Global Burden of Disease
GHSQ: general help seeking questionnaire
GP: general practitioner
ICCMH: Integrated Clinical and Community Mental Health
IPQ: Illness Perception Questionnaire
MDD: major depressive disorder
SD: standard deviation
SPSS: statistical package for social science
UoG: University of Gondar
YLDs: years lived with disability
WHO: World Health Organization
